# N-3 Polyunsaturated Fatty Acid Dehydrogenase Fat-1 Regulates Mitochondrial Energy Metabolism by Altering DNA Methylation in Isolated Cells of Transgenic Cattle

**DOI:** 10.3389/fmolb.2022.857491

**Published:** 2022-04-19

**Authors:** Xueqiao Wang, Lin Zhu, Zhuying Wei, Mingjuan Gu, Miaomiao Yang, Xinyu Zhou, Chunling Bai, Guanghua Su, Xuefei Liu, Lei Yang, Guangpeng Li

**Affiliations:** ^1^ State Key Laboratory of Reproductive Regulation and Breeding of Grassland Livestock, Inner Mongolia University, Hohhot, China; ^2^ School of Life Science, Inner Mongolia University, Hohhot, China

**Keywords:** *fat-1*, metabolism, rate-limiting enzyme, ATP, DNA methylation

## Abstract

The fatty acid dehydrogenase *fat-1* gene, derived from *Caenorhabditis elegans*, encodes n-3 polyunsaturated fatty acid dehydrogenase (Δ15 desaturase) and catalyzes the 18–20-carbon n-6 polyunsaturated fatty acids (n-6 PUFA) to generate corresponding n-3 polyunsaturated fatty acids (n-3 PUFA). Subsequently, fat-1 can influence the n-6: n-3 PUFA ratio in *fat-1* transgenic cells. This study aimed to explore which processes of energy metabolism are affected exogenous *fat-1* transgene and the relationship between these effects and DNA methylation. Compared with the wild-type group, the n-3 PUFA content in *fat-1* transgenic bovine fetal fibroblasts was significantly increased, and the n-6 PUFA content and the n-6: n-3 PUFA ratio decreased. In the context of energy metabolism, the increase of exogenous *fat-1* transgene decreased ATP synthesis by 39% and reduced the activity and expression of key rate-limiting enzymes in glycolysis, the tricarboxylic acid cycle, and oxidative phosphorylation, thus weakening the cells’ capacity for ATP production. DNA methylation sequencing indicated that this inhibition of gene expression may be due to altered DNA methylation that regulates cell energy metabolism. Exogenous *fat-1* transgenic cells showed changes in the degree of methylation in the promoter region of genes related to energy metabolism rate-limiting enzymes. We suggest that alters the balance of n-6/n-3 PUFA could regulate altered DNA methylation that affect mitochondrial energy metabolism.

## Introduction

In mammals, the n-3 polyunsaturated fatty acid (PUFA) α-linolenic acid and n-6 PUFA linoleic acid cannot be synthesized *de novo* and are thus considered “essential dietary fatty acids”. Once ingested, these PUFA are then catalyzed by desaturase and elongase enzymes (Wallis et al., 2002; Mokoena et al., 2020). The n-3 desaturase gene (*fat-1*) encodes n-3 fatty acid desaturase in the *Caenorhabditis elegans* genome (Spychalla et al., 1997; Wang et al., 2006), catalyzes n-6 PUFA to yield corresponding n-3 PUFA and subsequently influences the *in vivo* ratio of n-6 PUFA to n-3 PUFA (Mokoena et al., 2020). In lipid and fatty acid metabolism, PUFA can not only inhibit fat cell differentiation but also promote fatty acid oxidation (Kim and Voy. 2021). Additionally, PUFA can be used to treat non-alcoholic steatohepatitis by targeting the metabolism of liver to encourage mitochondrial β-oxidation (Okada et al., 2018). Another study showed that the synergistic effect of n-3 PUFA and squalene, a polyunsaturated hydrocarbon to maintain the antioxidant status of mitochondria. Squalene and n-3 PUFA work to regulate the activity of mitochondrial energy metabolism-related enzymes, thereby resisting reactive oxygen species in the liver mitochondria of older rats (Obulesu et al., 2014). In mammals, *fat-1* transgenic with not only have anti-arrhythmic effects (Kang, 2005), anti-tumor effects (Nowak et al., 2007), immune effects (Yamashita et al., 2013), and anti-inflammatory effects (Tomio et al., 2013) but also increased cholesterol metabolism (Kim et al., 2012) and nerve damage repair (Gladman et al., 2012). In livestock animals (e.g., sheep and cows), we produced *fat-1* genetically modified animals and found that this increase in endogenous n-3 PUFA caused by *fat-1* transgene not only changed the metabolic pathways of transgenic animals, but also changed the intestinal flora to benefit animal health (Duan et al., 2012; Wu et al., 2012; Liu et al., 2015).

The impact of changes in PUFA on metabolism has been examined in several previous studies, and its impact in the field of epigenetics has also received extensive attention. Daily supplementation of docosahexaenoic acid (DHA) for pregnant women at 18–22 weeks of gestation has increased global methylation levels (Lee et al., 2013). The maternal intake of n-3 PUFA during pregnancy may influence the DNA methylation status of the offspring as well (Bianchi et al., 2019). Other studies have shown significant changes in DNA methylation in skeletal muscle after bariatric surgery (Barres et al., 2013). Additionally, DNA methylation alters the expression of different genes involved in energy metabolism, such as insulin genes in glucose homeostasis (Kuroda et al., 2009), the glucose transporter 4 (GLUT4)-encoding gene (Yokomori et al., 1999), and the peroxisome proliferator activation receptor γ gene (PPARγ) (Fujiki et al., 2009). Real-time quantitative polymerase chain reaction (RT-qPCR) results have shown that the expressions of four oxidative phosphorylation (OXPHOS)-related genes were significantly downregulated in islet cells from patients with abnormal glucose metabolism (Olsson et al., 2011). In skeletal muscle, DNA methylations in genes of *NDUFA5*, *COX11*, and *ATP6V1H* significantly affected transcriptions of these genes (Ling et al., 2007; Ronn et al., 2008; Ronn et al., 2009). Interestingly, in gastric cancer cells, DNA methylation also affects PUFA biosynthesis (Lee et al., 2020). These studies indicate that DNA methylation plays an important role in energy metabolism regulation, whether in fat cells, muscle cells, or pancreatic islet cells. However, the degree and direction of DNA methylation varies in different cells.

The effect of exogenous gene transfer on DNA methylation has been studied before (Fang et al., 2014). In this study, bovine fetal fibroblasts (bEFs) isolated from *fat-1* transgenic (FT) and wild-type, (WT) cattle were used to detect the activity, expression, and product content of rate-limiting enzymes associated with energy metabolism. Specifically, the methylation pattern in promoter regions of rate-limiting enzyme-encoding genes were analyzed to determine the effects exogenous *fat-1* transgene may exert on bovine metabolism.

## Materials and Methods

### Ethics Statement

All experimental procedures in this study were consistent with the National Research Council Guide for the Care and Use of Laboratory Animals. All protocols were approved by the Institutional Animal Care and Use Committee at Inner Mongolia University.

### Animals

Three *fat-1* transgenic cattle fetuses (FT_1, FT_2 and FT_3) and three wild-type Luxi cattle fetuses (WT_1, WT_2 and WT_3) at 50–60 days were obtained from Shengmu Experimental Base (Holinger District, Hohhot). The fetuses were rinsed with normal saline, placed in phosphate-buffered saline (PBS) containing dual antibodies (15140-122, Gibco, United States), placed in an icebox, and brought back to the laboratory for analysis.

### Cell Isolation and Culture

The head, limbs, internal organs, and bones of the fetus were removed and discarded. The remaining tissues were rinsed three times with PBS and then once with 75% ethanol for 15 s and transferred back to PBS. Then, the samples were sectioned into 1–3-mm slices and placed in Petri dishes. collagenase IV at a concentration of 1 mg/ml was added; cultures were maintained at 38.5°C with 5% CO_2_ for 2–3 h. After the tissues were dissolved, the mixtures were centrifuged at 1,500 rpm/min for 5 min. The cell pellet was collected, resuspended in DMEM F12 (11330107, Gibco, United States) supplemented with 20% FBS (10099141, Gibco, United States), and cultured in 60-mm dishes at 38.5°C with 5% CO_2_. bEFs were passaged when they reached 90% confluence.

### Fatty Acid Gas Chromatography

Fatty acids were analyzed from bEFs when they reached 80% confluency. Briefly, bEFs were collected using 0.05% trypsin (25300062, Gibco, United States). Digested cells were placed into PBS-containing centrifuge tubes and centrifuged at 1,500 rpm for 5 min. Next, 1 ml 2.5% H_2_SO_4_/methanol solution was added to the isolated supernatant, which was then incubated in an 80°C water bath for 90 min. The solution was allowed to cool to room temperature before 1.5 ml 0.9% NaCl solution and 1 ml n-hexane was added. The solution was vigorously mixed for 5 min and then centrifuged at 1,500 rpm for 5 min. The organic phase was transferred to a new centrifuge tube. Saturated KOH-methanol solution was added, vigorously mixed for 5 min, and centrifuged at 1,500 rpm for 10 min. The upper liquid was collected into gas chromatography–mass spectrometry (GC-MS) sample vials for fatty acid analysis. The gas chromatographic conditions were as follows: carrier gas helium, chromatographic column HP-88, constant linear velocity 20.0 cm/s, separation ratio 20.0%, injection volume 1 μL, heating program 60°C for 1 min, 40°C/min heating to 140°C, maintained at 140°C for 10 min, increased to 240°C at 4°C/min, and maintained at 240°C for 15 min.

### RNA-Seq of bEFs

Total RNA of bEFs was extracted using Trizol reagent (15596026, Invitrogen, United States). Poly (A) RNA was purified from total RNA (5 μg) by poly-T oligo-attached magnetic beads and then broken into small fragments. The spliced RNA fragments were reverse-transcribed to construct the final cDNA library. Paired-end sequencing was performed in LC Science using an HiSeq 4000 (Illumina, United States). The read values of the samples were aligned with the UCSC (http://genome.ucsc.edu/) reference genome using the HISAT package. This package initially removed a portion of the reads based on the quality information accompanying each read value, and then mapped the reads to the reference genome. All the transcripts in the samples were combined to reconstruct a comprehensive transcriptome using Perl Scripts. StringTie and edgeR were used to estimate expression levels of all transcripts. The differentially expressed mRNAs and genes with log2 (fold change) > 1 or log2 (fold change) < −1 and with statistical significance (*p* value <0.05) were screened using R.

### Metabolic Substrates and Enzymes Assays

The activity of key restriction enzymes and substrate contents in glycolysis, TCA cycle, and OXPHOS were measured using commercial kits following the manufacturer’s instructions. The following commercial kits, all obtained from Comibio Biotechnology, China: the content of ATP (ATP-1-Y), glucose (PT-1-Y), FDP (FDP-1-G), PA (PA-1-Y), CA (CA-1-Y), NADH (NAD-1-Y) and the activity of HK (HK-1-Y), PFK (PFK-1-Y), PK (PK-1-Y), ICDHm (ICDHm-1-Y), α-KGDH (KGDH-1-Y), SDH (SDH-1-Y), MDHm (MDHm-1-Y), mitochondrial complex I (FHTA-1-Y), complex III (FHTC-1-Y), complex IV (FHTD-1-Y), complex V (FHTE-1-Y). Briefly, 10^7^ cells were placed in a homogenizing tube containing 1 ml kit extract with ∼15 ceramic beads. The solution was homogenized at a low temperature with a homogenizer (Bertin, France). The supernatant was then added to a 96-well plate along with other reagents, as instructed. The absorbance was recorded with a microplate spectrophotometer (Thermo, United States) to calculate the enzyme activity or metabolite concentration.

### Real-Time Quantitative PCR Assay

Total RNA was isolated from tissues using the RNAiso Plus kit (9109, Takara, Japan) according to the manufacturer’s instructions. Briefly, RNA was then reverse-transcribed into cDNA using a cDNA reverse transcription kit (RR036A, Takara, Japan). RT-qPCR was performed using a real-time PCR detection system (7500, ABI, United States). The reaction mixture (20 μL) contained 1 μL cDNA, 0.5 μL of each primer, and 10 μL TB Green Supermix (RR820A, Takara, Japan). The primers used are listed in Supplementary Table S1. The protocol was as follows: initial denaturation step at 95°C for 30 s and then 40 cycles of [95°C for 15 s, 60°C for 30 s]. Relative abundance was quantified using the 2^−ΔΔCt^ method. *RPLP0* was used as a housekeeping gene.

### Genome-wide DNA Methylation Analysis

Total DNA was extracted using a QIAamp Fast DNA Tissue Kit (51404, Qiagen, Germany) following the manufacturer’s protocol and quantified using a spectrophotometer and the A260/A280 ratio. DNA samples were fragmented using sonication and subjected to bisulfite conversion. The Accel-NGS Methyl-Seq DNA Library Kit (Swift, MI, United States) was utilized for attaching adapters to single-stranded DNA fragments. Briefly, the Adaptase step is a highly efficient, proprietary reaction that simultaneously performs end repair, tailing of 3′ ends, and ligation of the first truncated adapter complement to 3′ ends. The Extension step incorporates the truncated adapter 1 by a primer extension reaction. The Ligation step is used to add the second truncated adapter to the bottom strand only. The Indexing PCR step increases the yield and incorporates full-length adapters. Bead-based Solid Phase Reverse Immobilization (SPRI) clean-ups are used to remove both oligonucleotides and small fragments, as well as to change enzymatic buffer composition. Finally, the pair-end 2 150 bp sequencing was performed in LC Science using an HiSeq 4000 (Illumina, United States).

### 5-mC and 5-hmC Quantitative Assays

DNA methylation and hydroxymethylation were quantified using the MethylFlash methylated and hydroxymethylated colorimetric DNA quantification kits (P-1034; P-1036; Epigentek, United States), respectively, according to the manufacturer’s instructions. The percentage of methylated DNA was proportional to the optical density intensity measured. All samples were obtained in triplicates.

### Western Blot Assay

BEFs were rinsed with PBS and lysed in 150 μL of ice-cold Radio Immunoprecipitation Assay buffer. Proteins were extracted from tissues with cell lysis buffer, boiled for 5 min, and stored at −80°C. Samples were separated using a 10% sodium dodecyl sulfate-polyacrylamide gel electrophoresis (SDS-PAGE), and then electrically transferred to polyvinylidene fluoride membranes blocked with 5% nonfat dry milk in TBS-Tween 20 (blocking buffer) for 1 h. The membrane was subsequently incubated with the following antibodies, all obtained from Santa Cruz Biotechnology, United States unless otherwise stated: anti-DNMT1 (1:500; sc-271729), anti-DNMT3a (1:500; sc-373905), anti-DNMT3b (1:500; sc-393279), anti-TET1 (1:500; sc-293186) and anti-TET2 (1:1,000; ab94580; Abcam, United States) and anti-TET3 (1:1,000; ab139311; Abcam, United States) or with a polyclonal antibody against α-Tubulin (11224-AP, proteinch, China) at 1:1,000 in TBST at 4°C overnight. Membranes were then washed three times and incubated with a horseradish peroxidase-conjugated goat anti-mouse and anti-rabbit secondary antibody at 1:5,000 in blocking buffer.

### Prediction of CpG Islands

The CpG islands and CpG sites in the promoters of *PKM*, *PFKM*, *IDH2*, *MDH2*, *NDUFS1* and *ATP5F1C* were examined using MethPrimer software online (http://www.urogene.org/cgi-bin/methprimer/methprimer.cgi).

### MeDIP Assay

The MeDIP analysis was performed according to the manufacturer’s instructions (ab117133; Abcam, United States). Sonication of total genomic DNA (three pulses for 10–12 s at level 2 with 30–40 s intervals between pulses while resting on ice) produced DNA fragments between 200 bp and 1,000 bp in length. Single-stranded DNA was produced by heating and denaturing 1 µg of DNA. Then, 1 μL of anti-5mC antibody (or normal mouse IgG for a negative control) was added to induce immunoprecipitation at room temperature for 2 h. Proteinase K was added and the mixture incubated at 65°C for 1 h to release mDNA from the DNA/antibody complex. Finally, the mDNA was captured, eluted, and detected by real-time quantitative PCR. The qPCR primers are listed in Supplementary Table S2.

### Statistical Analysis

GraphPad Prism 8.0 software was used for statistical analyses. The fatty acid contents of each tissue and the enzymatic activities and mRNA expressions in each group of cells were expressed as mean ± SEM. The mean of *fat-1* transgenic and wild-type cattle samples was compared by one-way analysis of variance (ANOVA). *p* < 0.05 was considered statistically significant.

## Results

### Exogenous *Fat-1* Transgene Affected ATP Synthesis Pathway

**FIGURE 1:**
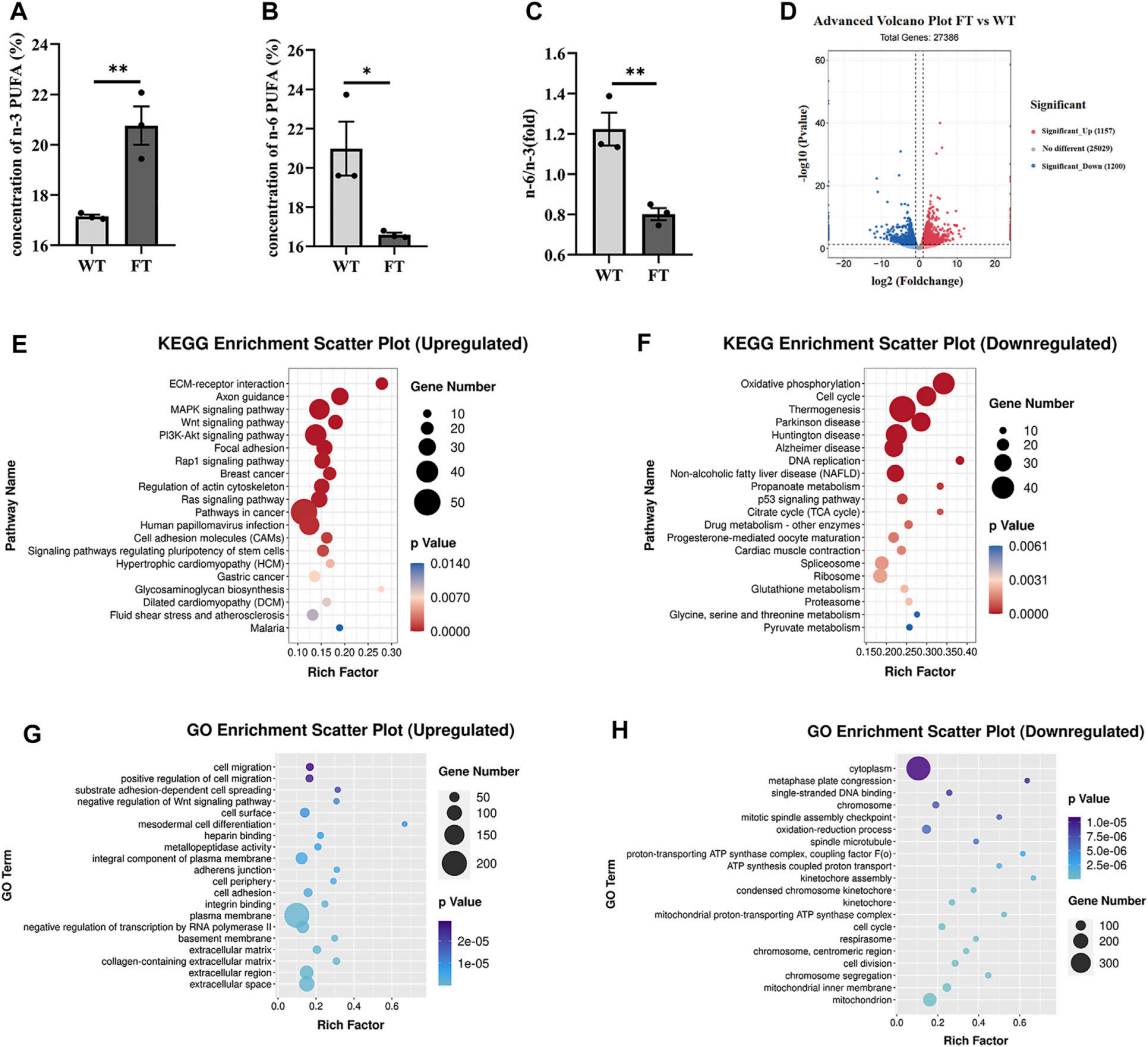
Exogenous *fat-1* gene affects ATP synthesis in bovine fetal fibroblasts (bEFs). **(A,B)** Percent total of n-3 PUFA and percent total of n-6 PUFA by Gas Chromatography-Mass Spectrometer (GC-MC). N-3 PUFA (sum of C18:3n3, C20:3n3, C20:5n3 C22:6n3 and C18:2n6t), N-6 PUFA (sum of C18:2n6c, C18:3n6, C20:3n6 and C20:4n6). (*n* = 3) **(C)** The ratio of n-6 PUFA to n-3 PUFA was statistically lower in FT. (*n* = 3) **(D)** Differential expression genes between fat-1 and control fibroblasts. Significant_Up: significantly upregulated genes; No differernt: no significant differences in gene expression; Significant_down: significantly downregulated genes. (*n* = 3) **(E)** KEGG pathway upregulating enrichment. **(F)** KEGG pathway downregulating enrichment. **(G)** GO upregulated enrichment analysis of differential genes. **(H)** GO upregulated enrichment analysis of differential genes. Each dot represents an independent experiment, 0001 Each dot represents an independent experiment **(A–C)**. All data are presented as mean ± SEM. Compared with the WT group, *p < 0.05, **p < 0.01, ***p < 0.001, ****p < 0.0001; t-tests were used to calculate the *p*-values.

Both DNA and mRNA of exogenous *fat-1* gene were significantly expressed in the transgenic bEFs, but not detected in WT cells (Supplementary Figure S1). n-3 PUFA were quantified using gas chromatography; % total n-3 PUFA (sum of C18:3n3, C20:3n3, C20:5n3 and C22:6n3) were significantly increased in FT groups in comparison to WT groups (17.149 ± 0.070 vs. 20.764 ± 0.761, *p* = 0.009, Figure 1A), while % total n-6 PUFA (sum of C18:2n6t, C18:2n6c, C18:3n6, C20:3n6 and C20:4n6) were significantly reduced in FT cells (20.976 ± 1.377 vs. 16.591 ± 0.102, *p* = 0.034, Figure 1B). Subsequently, the n-6/n-3 ratio was significantly lower in the FT group (Figure 1C). These results suggest the exogenous *fat-1* gene impacts n-6 and n-3 PUFA synthesis and subsequently alters the balance of n-6/n-3 PUFA.

RNA-seq analysis was performed on FT and WT cells. Out of a total of 27,386 differential genes, 1,157 genes were significantly up-regulated, and 1,200 genes were significantly down-regulated (Figure 1D). The KEGG pathway identified ECM-receptor interaction, axon guidance, and MAPK signaling pathways were significantly upregulated (Figure 1E). Among the pathways identified by the KEGG pathway that were significantly downregulated included OXPHOS, thermogenesis, and the TCA cycle (Figure 1F). GO enrichment analysis suggested the upregulated genes were mainly related to cell migration, positive regulation of cell migration, and substrate adhesion-dependent cell spreading (Figure 1G). Downregulated genes identified included enriched cytoplasm, oxidation-reduction process, single-stranded DNA binding. However, many genes related to ATP synthesis, oxidation-reduction process, and the proton-transporting ATP synthase complex were also downregulated (Figure 1H). These results suggest that the transgenic *fat-1* gene not only changes the fatty acid composition to decreased the rato of n-6: n-3 PUFA, but also regulates and influences energy metabolism, possibly through the ATP synthesis pathway.

### Exogenous *Fat-1* Transgene Inhibited Energy Metabolism and Reduced ATP Production

**FIGURE 2:**
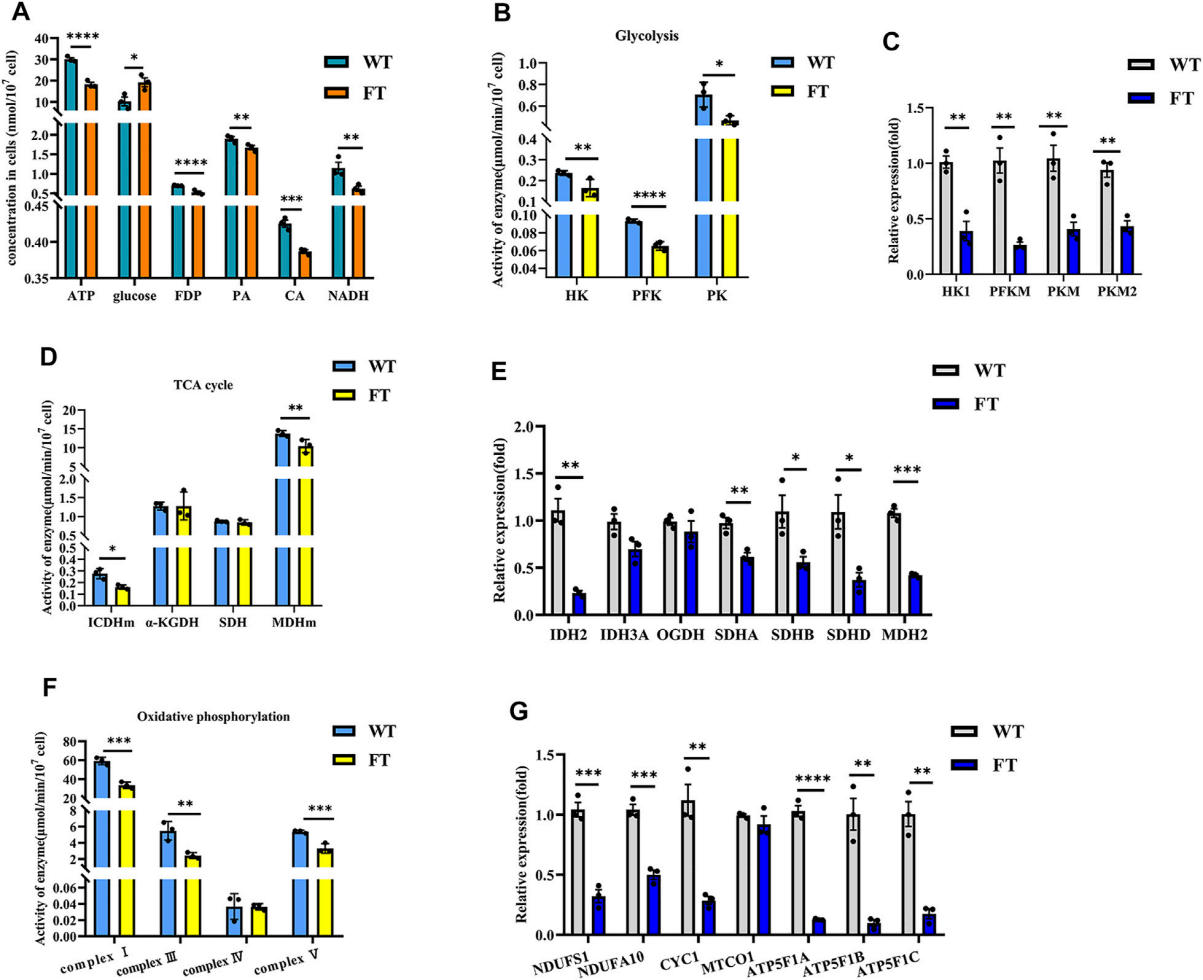
Exogenous *fat-1* gene is effect on bEFs energy metabolism pathway of cattle. **(A)** Energy metabolism related product contents (*n* = 3). **(B,D,F)** Activity for key rate-limiting enzymes involved in glycolysis **(B)** and TCA cycle **(D)** and OXPHOS **(F)** pathway. (*n* = 3). **(C, (E,G)** Expression of mRNA for key rate-limiting enzymes involved in glycolysis **(C)** and TCA cycle **(E)** and OXPHOS **(G)** pathway by RT-qPCR in FT and WT bEFs. (*n* = 3) All assays involving enzyme activity and metabolite concents were statistically calculated by calculating the absorbance in microplate spectrophotometer. Each dot represents an independent experiment. All data are presented as mean ± SEM. Compared with the WT group, *p < 0.05, **p < 0.01, ***p < 0.001, ****p < 0.0001; t-tests were used to calculate the *p*-values.

ATP levels in FT and WT cells decreased by approximately 39% (30.101 ± 0.599 vs. 18.318 ± 0.762 nmol/min/10^7^ cells, *p* < 0.0001, Figure 2A), while glucose content increased by 47% (10.265 ± 1.633 vs. 19.347 ± 3.207 μmol/10^7^ cells, *p* = 0.032, Figure 2A). The activity of key glycolytic rate-limiting enzymes hexokinase (HK), phosphofructokinase (PFK) and pyruvate kinase (PK) in FT cells were significantly lower than those in WT cells (Figure 2B). The catalytic product of PFK, fructose-1,6-diphosphate (FDP) was also significantly reduced (0.700 ± 0.011 vs. 0.528 ± 0.021 mg/10^7^ cells, *p* < 0.0001, Figure 2A). Pyruvate (PA), a product of PK, was also significantly lower in FT cells (1.897 ± 0.039 vs. 1.673 ± 0.046 μg/10^7^ cell, *p* = 0.004, Figure 2A). RT-qPCR further confirmed that the mRNA expression levels of *HK1*, *PFKM*, and *PKM* subunits were significantly down-regulated (Figure 2C).

Detection of citric acid (CA) and NADH, products of the TCA cycle, were significantly lower in FT cells (0.425 ± 0.008 vs. 0.387 ± 0.002 nmol/10^7^ cells, *p =* 0.0009 and 1.151 ± 0.104 vs. 0.620 ± 0.056 nmol/10^7^ cells, *p* = 0.001, Figure 2A). In FT cells, the activity of mitochondrial isocitrate dehydrogenase (ICDHm) and mitochondrial malate dehydrogenase (MDHm), key rate-limiting enzymes in the TCA cycle, were significantly reduced in FT cells when compared to WT (Figure 2D). RT-qPCR results also confirmed that *IDH2* of ICDHm and *MDH2* were significantly downregulated (Figure 2E). There was no significant difference between the activity of α-ketoglutarate (α-KGDH) (Figure 2D) and the expression of *OGDH* encoding it (Figure 2E). Although there was no significant change in succinate dehydrogenase (SDH) activity (Figure 2D), the expressions of *SDHA*, *SDHB* and *SDHD* were downregulated to varying degrees (Figure 2E).

NADH dehydrogenase (complex I), pigment reductase (complex III), cytochrome oxidase (complex IV), and ATP synthase (complex V) participate in OXPHOS. Subsequently, these enzymes were examined and found (with the exception of complex IV) to be significantly downregulated (Figure 2F), which was additionally confirmed by RT-qPCR (Figure 2G).

### Genome-wide DNA Methylation Is Elevated in *Fat-1* Transgenic bEFs

**FIGURE 3:**
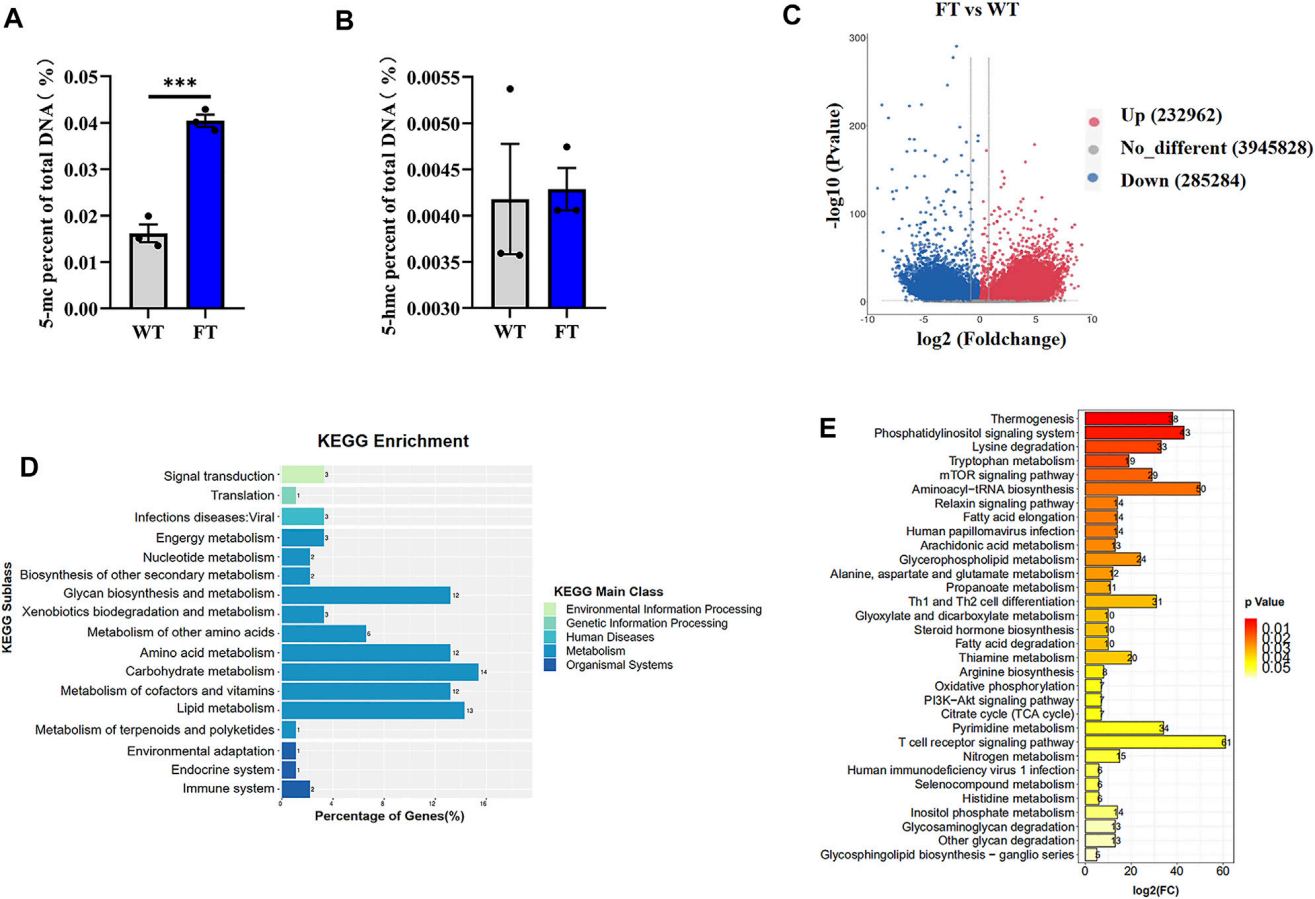
DNA methylation is altered in *fat-1* transgenic cells. **(A,B)** Content of 5-mC and 5-hmC were quantified using the MethylFlash methylated and hydroxymethylated colorimetric DNA quantification kits. Each dot represents an independent experiment. (*n* = 3). **(C)** Methylation differences between *fat-1* and control fibroblasts. In the plotted volcano plot, red dots represent significantly hypermethylated sites, blue dots represent significantly hypomethylated sites, and grey represent no significant difference. **(D)** KEGG pathway enrichment analysis. **(E)** KEGG classification enrichment analysis. All data are presented as mean ± SEM. Compared with the WT group, *p < 0.05, **p < 0.01, ***p < 0.001, ****p < 0.0001; t-tests were used to calculate the *p*-values.

Previous studies have shown exogenous addition of n-3 PUFA increased the overall degree of DNA methylation in adipocytes (Perfilyev et al., 2017). Therefore, we performed global methylation on FT cells compared to WT cells. Higher levels of % total DNA methylation was observed in FT cells in comparison to WT (Figures 3A,B), although levels of 5-hmc methylation was not statistically significant. Genome-wide DNA-methylation analysis also identified differences between the two groups (Figure 3C). KEGG analysis revealed that approximately 80% of the differential genes were enriched in metabolic pathways, and significant changes occurred in DNA methylation of genes related to thermogenesis, fatty acid elongation, TCA cycle, and OXPHOS between FT and WT cells (Figures 3D,E).

### DNA Methylation in the Promoter Region of Rate-Limiting Enzymes Are Elevated in *Fat-1* Transgenic bEFs

**FIGURE 4:**
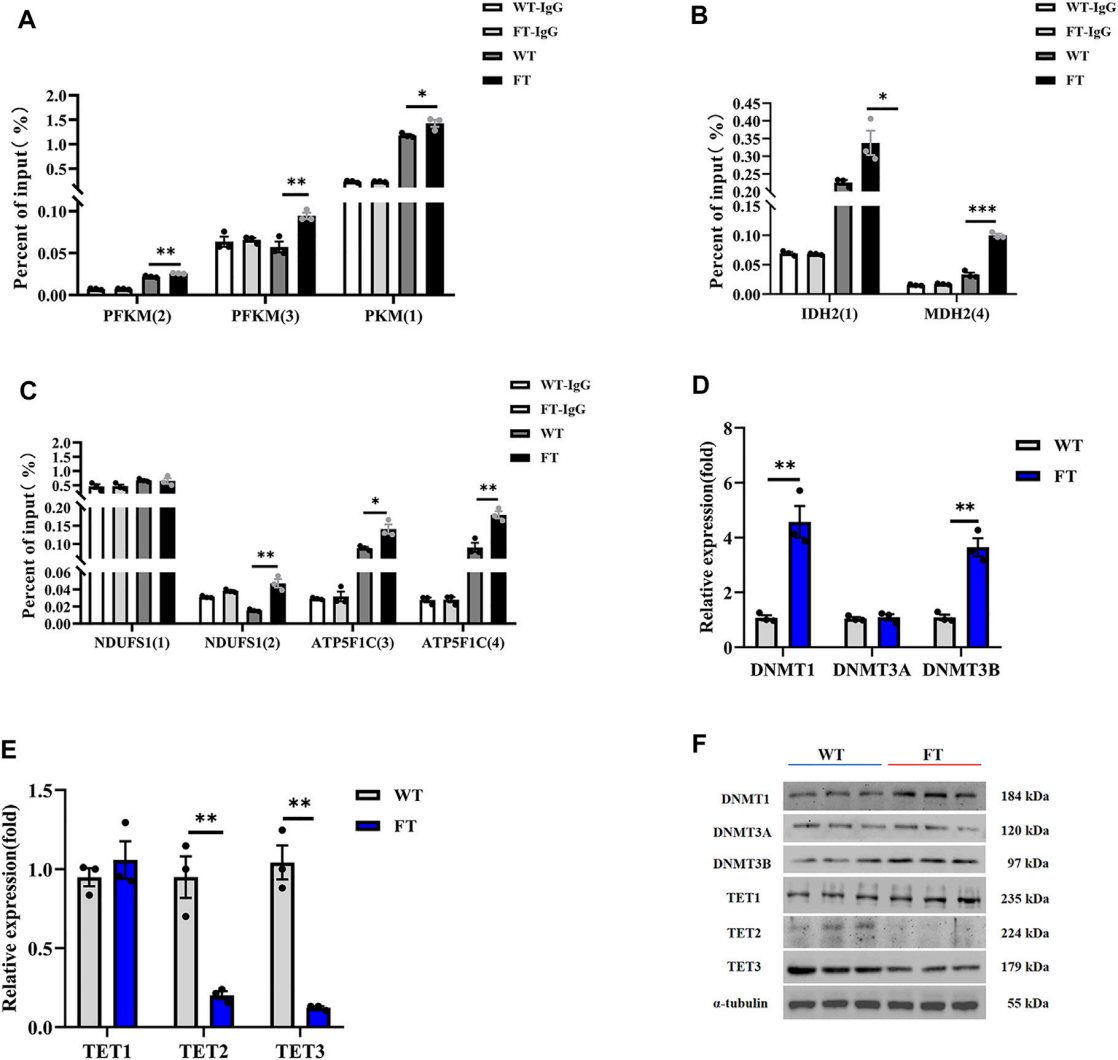
Effect of DNA methylation on rate-limiting enzymes related to energy metabolism. **(A–C)** Methylation levels in promoters of genes associated with rate-limiting enzymes in glycolysis **(A)**, TCA cycle **(B)**, and OXPHOS **(C)** (*n* = 3). The CpG island was predicted in the promoter region of the gene, and then the predicted CpG island was detected by Methylated DNA immunoprecipitation (MeDIP) by RT-qPCR. Location of CpG island was shown in Supplementary Figure S2. **(D,E)** Expression of mRNA for methyltransferases and demethyltransferases by RT-qPCR in WT and FT bEFs. (*n* = 3). **(F)** Expression of protein for methyltransferases and demethyltransferases by immunoprecipitation in WT and FT bEFs. Western blots obtained by immunoblotting antibodies directed against DNMT1, DNMT3A, DNMT3B, TET1, TET2 and TET3. WT (blue line) vs. FT (red line). indicates the location of Island, please refer to Supplementary Figure S2 for detailed location. e g., PFK (2) means PFK (Island 2). Each dot represents an independent experiment. All data are presented as mean ± SEM. Compared with the WT group, *p < 0.05, **p < 0.01, ***p < 0.001, ****p < 0.0001; t-tests were used to calculate the *p*-values.

Methylation of genes associated with the key limiting enzymes in glycolysis, TCA cycle, and OXPHOS were all significantly increased in FT cells compared with WT cells (Figure 4A). *PFKM* and *PKM* in glycolysis, *IDH2* and *MDH2* in the TCA cycle, and *NDUFS1* and *ATP5F1* in OXPHOS were all hypermethylated at their promoters’ CpG island in FT cells (Figures 4A–C), correlating to *fat-1* transgene. This suggests balance of n-6: n-3 PUFA ratios could play a role in energy metabolism-associated gene expression. We then analyzed activity of the methylases DNMT1, DNMT3A, DNMT3B, TET1, TET2, and TET3. As expected, expression of DNMT1 and DNMT3B increased, expression of TET2 and TET3 significantly decreased, and expression DNMT3A and TET1 did not change (Figures 4D–F). These results further suggest that exogenous *fat-1* transgene in the FT group can influence hypermethylation of genes related to energy metabolic rate-limiting enzymes by upregulating the methyltransferases DNMT1 and DNMT3B and downregulating TET2 and TET3 to reduce the demethylation of genes encoding these enzymes.

## Discussion

As an n-3 PUFA desaturase, *fat-1* transgenes have been shown to significantly alter ratio of n-6: n-3 PUFA by converting n-6 PUFA to n-3 PUFA in mice (Kang. 2007), pigs (Pan et al., 2010; Tang et al., 2014; Liu et al., 2016), cows (Wu et al., 2012), sheep (Duan et al., 2012), and zebrafish (Sun et al., 2020). A similar phenomenon was also observed in this study. This study focused on the n-3 PUFA dehydrogenase gene *fat-1*, which affects the substrates of n-3 PUFA and n-6 PUFA, thereby affecting the ratio of n-6: n-3 PUFA, as to which changes in the effects of two fatty acids on energy metabolism will be studied in the future. Exactly which of the two fatty acids plays a greater role cannot be determined. Interestingly, Previous studies have shown that fish oil supplementation, rich in n-3 PUFA, can increase glucose production in human liver cells (Aggarwal et al., 2018). Furthermore, n-3 PUFA participate in the metabolic pathways of mitochondrial respiration and energy production. In diabetic patients, n-3 PUFA can improve impaired energy metabolism. Long-term supplementation of n-3 PUFA in diabetic patients has been associated with improved energy metabolism in the brain and myocardium mitochondria, suggesting n-3 PUFA supplementation can prevent cardiovascular or neurological complications in the later stage of diabetes (Ochoa et al., 2005; Carvalho-Silva et al., 2019). In this way, changes in n-3 PUFA content may have more effects on the body. Studies have shown that *fat-1* transgenes regulate energy metabolism by downregulating the ratio of n-6: n-3 PUFA (Hagopian et al., 2010; Sun et al., 2020). Moreover, PUFA act as a substrate for energy metabolism and signaling molecules, which can also regulate gene expression (Jump. 2004; Jump et al., 2008). Romanatto et al. found that *fat-1* mouse tissues was associated with better glucose homeostasis (Romanatto et al., 2014). The complete oxidative decomposition of glucoses starts during glycolysis, in which HK, PFK, and PK are the key rate-limiting enzymes. The activity of these enzymes affected the rate and direction of glycolysis (Tappy et al., 2006). In this study, glucose content and the activity and expressions of HK, PFK and PK of *fat-1* transgenic cells were significantly decreased when compared to the control group. FDP, catalytic product of PFK (Pinheiro et al., 2010) and PA, the final product of glycolysis, was also significantly reduced in FT cells. These results suggest that the change of n-6: n-3 PUFA ratios could inhibit glucose utilization during glycolysis in FT bEFs.

Obulesu et al. reported dietary supplementation with squalene and PUFA significantly attenuated age-associated inhibition on mitochondrial TCA cycle enzyme activity, including ICDHm, α-KGDH, SDH, MDH, and mitochondrial complex I, which maintained hepatic energy status at near normal levels (Obulesu et al., 2014). In *fat-1* transgenic mice, activity of complex I was weakened, and the products of the TCA cycle also decreased (Sun et al., 2020). The present data indicated that, in the *fat-1* transgene cells, products from glycolysis (glucose, PA and CA), the key limiting enzymes in TCA cycle (IDHm, SDH, and MDHm), and key enzymes in OXPHOS were all downregulated. These results indicated that three main processes of energy metabolisms, glycolysis, the TCA cycle and OXPHOS were all inhibited in FT cells, suggesting *fat-1* transgene attenuates energy metabolism by weakening the activity of the key-limiting enzymes related to metabolism.

The methylation mark 5mC is often associated with gene repression within gene promoters (Laird. 2010; Pawson. 2004). In this study, 5mC was significantly increased in exogenous *fat-1* transgenic bEFs, indicating increased genome-wide methylation levels, which It may be the reason for the down-regulation of the gene expression of key rate-limiting enzymes in energy metabolism. In cancer cells, α-ketoglutarate increases the content of 5hmC and down-regulates the content of 5mC (Lin et al., 2015). Our results show that there is no significant difference in the content of 5hmC. Changes, which led us to notice that in Figure 2D, the activity of α-ketoglutarate dehydrogenase did not differ between the two groups. This data may be related to 5hmC. Since this study focuses more on the fact that the exogenous *fat-1* transgene inhibits the expression of key rate-limiting enzymes in energy metabolism through DNA methylation, for demethylation-related 5hmC or 5fC or 5caC, we will in follow-up research. In a study of *fat-1* transgenic sheep, we reported that hypermethylation of the expression vector promoter region resulted in silencing of the target genes (Duan et al., 2012). We further found in transgenic sheep that exogenous *fat-1* expression was influenced by the methylation status of 721-1346nt, regulated by the methylation level of CAG promoters 101, 108 and 115nt, and maintained by DNMT1 for hypermethylation (Yang et al., 2017). Dietary fat quality impacts genome-wide DNA methylation patterns, as demonstrated in a cross-sectional study of Greek preadolescents (Voisin et al., 2015). In addition, mice fed with α-linolenic acid had 1–2% higher average methylation levels of fatty acid dehydrogenase 2 promoter in liver tissue (Niculescu et al., 2013). *In vitro*, following treatment of HT29/219 cancer cells with 100 μM DHA and EPA for 6 days, the overall methylation levels was significantly increased, particularly from higher expression of DNMTs (Sarabi and Naghibalhossaini. 2018). Other studies have compared PUFA intake and genome-wide methylation and found increasing levels of dietary PUFA was associated with an increase in DNA methylation (Aslibekyan et al., 2014; Gonzalez-Becerra et al., 2019). In this study, the *fat-1* transgene decreased the ratio of n-6: n-3 PUFA and increased the overall degree of genome-wide methylation. Methylation of important rate-limiting enzymes involved in glycolysis, the TCA cycle and OXPHOS, which are involved in ATP production synthesis, were significantly increased in FT cells, suggesting that energy metabolism and ATP production were reduced. Compared with WT group, expressions of DNA methyltransferase DNMT1 and DNMT3B was significantly upregulated in FT cells, while expressions of demethylases TET2 and TET3 were significantly decreased. Our results suggest that changes in DNA methylation of the rate-limiting enzymes related to energy metabolism could be caused by DNA methyltransferase. Further studies will investigate how *fat-1* regulates DNA methylation through methyltransferase.

## Conclusion

In conclusion, the change of n-6: n-3 PUFA ratios in *fat-1* transgenic cells lead to an increase in the methyltransferases DNMT1 and DNMT3B and a decrease in the demethylases TET2 and TET3. Therefore, glycolysis, the TCA cycle, and OXPHOS are impacted by *fat-1* transgene and gene methylation.

## Data Availability

The original contributions presented in the study are publicly available. This data can be found here: https://www.ncbi.nlm.nih.gov/geo/, GSE189689; GSE190293.

## Abbreviations

FT, *fat-1* transgenic; WT, Wild-type; PUFA, Polyunsaturated fatty acid; TCA cycle, Tricarboxylic acid cycle; OXPHOS, Oxidative phosphorylation; GLUT4, The glucose transporter 4; PPARγ, Peroxisome proliferator activation receptor γ; bEFs: Bovine fetal fibroblast cells; GC-MS, Gas Chromatography-Mass Spectrometer; RIPA, Radio Immunoprecipitation Assay; MeDIP, Methylated DNA immunoprecipitation; HK, hexokinase; PFK, Phosphofructokinase; PK, Pyruvate kinas; FDP, Fructose 1, 6-diphosphate; PA, Pyruvate; CA, Citric acid; ICDHm: Mitochondrial isocitrate dehydrogenase; MDHm, Mitochondrial malate dehydrogenase; α-KGDH, α-ketoglutarate; SDH, Succinate dehydrogenase.
