# Supplementary material for: N-3 Polyunsaturated Fatty Acid Dehydrogenase Fat-1 Regulates Mitochondrial Energy Metabolism by Altering DNA Methylation in Isolated Cells of Transgenic Cattle
Source: Front Mol Biosci. 2022 Apr 19;9:857491. doi: 10.3389/fmolb.2022.857491 (PMC9061993; doi:10.3389/fmolb.2022.857491)
Supplement: Supplementary file 1 [file DataSheet1.docx]

Supplementary Material

# Supplementary Figures and Tables

## Supplementary Figures


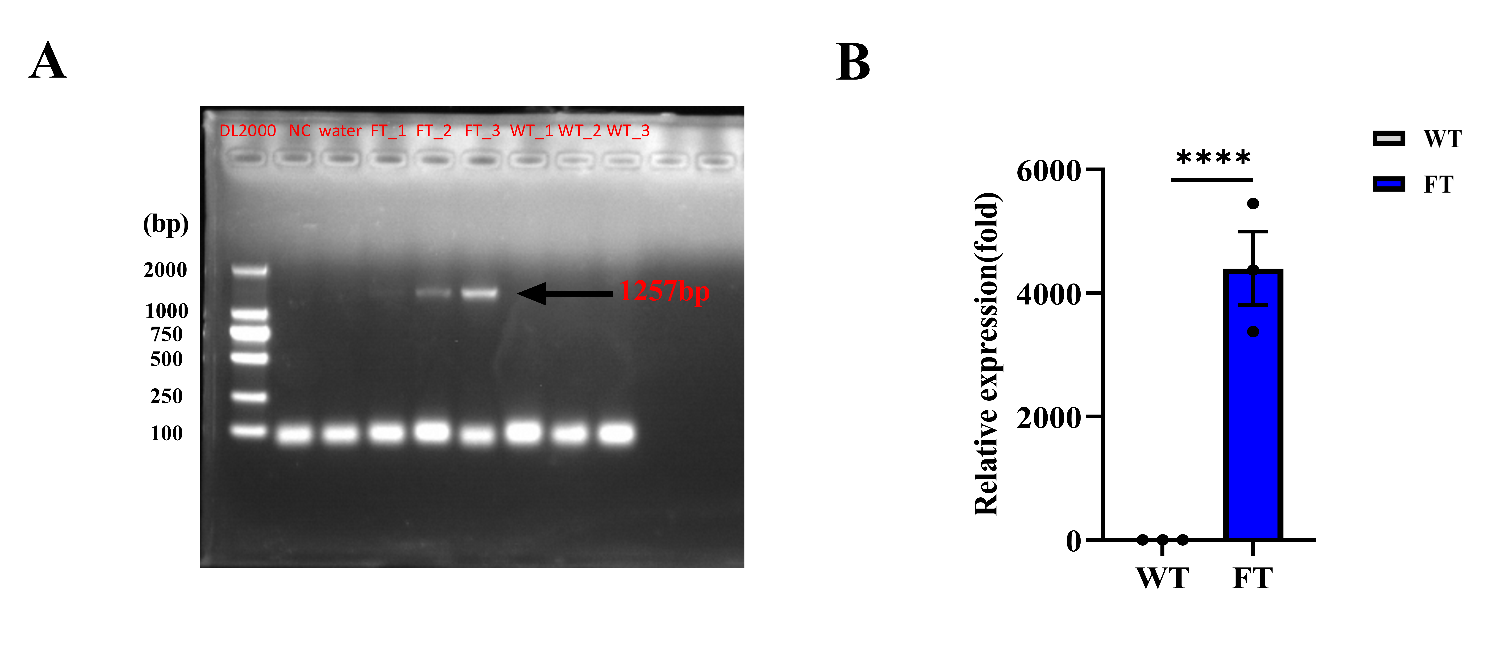


**Supplementary Figure 1.** Detection of fat-1 transgene in transgenic and wild-type cells. (A) DNA expression level (n=3). (B) mRNA expression level (n=3). Each dot represents an independent experiment. All data are presented as mean ± SEM. Compared with the WT group, **** *p*<0.0001; t-tests were used to calculate the *p*-values. Abbreviations: NC, negative control. Water as blank control.


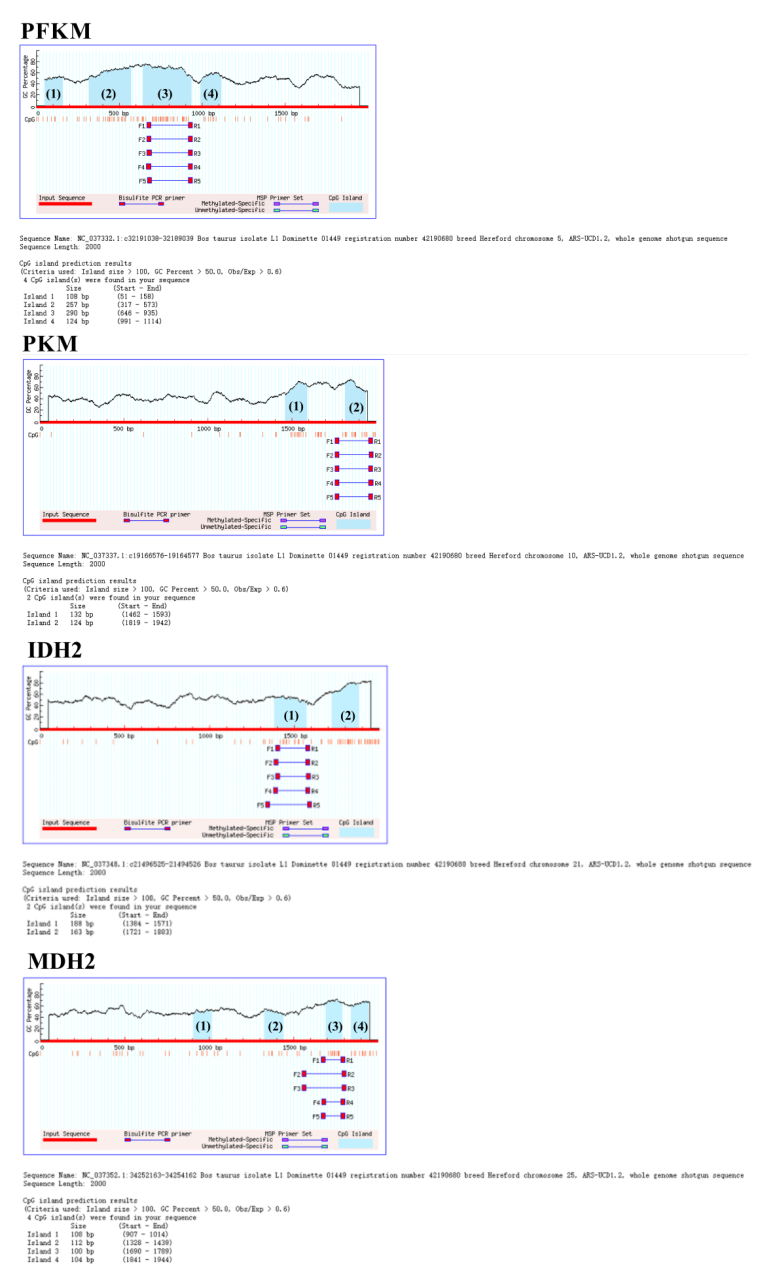

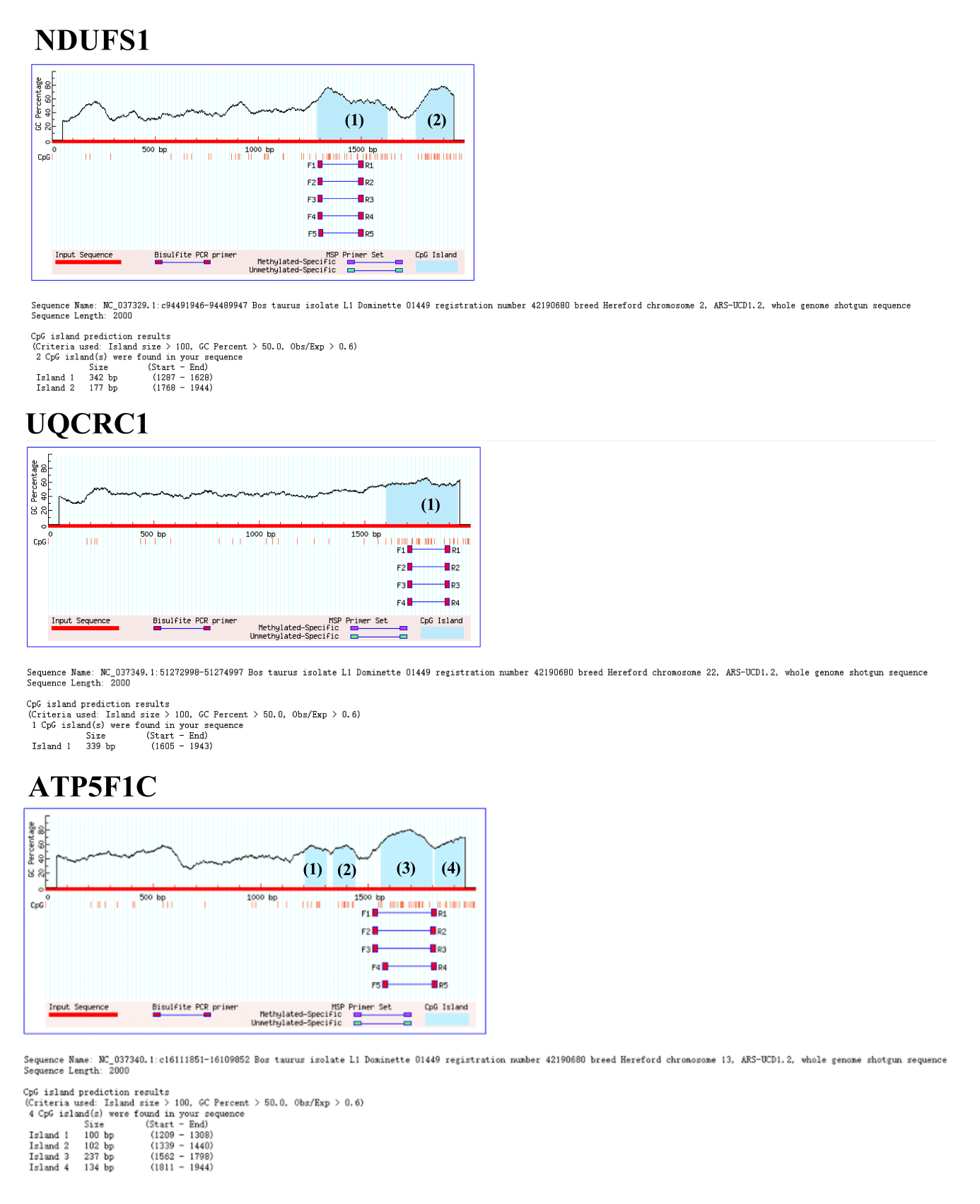


**Supplementary Figure 2.** Supplementary Figure 2. Prediction of DNA promoter methylation. (1) means Island 1, (2) (3) (4) has the same meaning. The article showed a subset of the predicted CpG islands of the genes that can be detected by RT-qPCR.

## Supplementary Tables

Supplementary Table 1. The primers of qPCR used in this study

| Gene name | Primer sequences | |
| --- | --- | --- |
|  | Sense (5’to 3’) | Anti-sense (5’to 3’) |
| *HK1* | TGAAGGAACTGTCACCAAAG | AAGAGAGAAGTGCTGGAAGG |
| *PFKM* | GGACAATCTGCAAAGAAGCCC | CCACCAGAGGTTAACACGGC |
| *PKM1* | CAAGCTGTTTGAAGAACTTG | AACTATCAAAGCTGCTGCTA |
| *PKM2* | TGCAGCACCTGATTGCCCGT | AATGGGTGACAGGCGACGGA |
| *IDH2* | AGATGGACGGCGATGAGATGAC | ATCATTGGTCTGGTCACGGTTC |
| *IDH3A* | GCAAATGTCCGACCATGTGTCTC | GAACCGACTCGAAGATTGCAACTC |
| *OGDH* | CCCATCCCAGTTTGATGTTC | ACCGATTCAAAGATGGCAAC |
| *MDH2* | AACAATGCTAAAGTAGCCGTGC | ATCTCGGGTCATACCTGGTTTT |
| *SDHA* | TGCAGACCATCTACGGAGCGGA | ACGTAGGAGAGCGTGTGCTTCCTCC |
| *SDHB* | AACTGTGGTCCTATGGTGCTG | CACATACATGTGTGGCAGAGG |
| *SDHD* | GTCCTATGGTGCTGGATGCT | CTTTCGGCTCTTGAGGACTG |
| *NDUFS1* | TTAGCAAATCACCCACTGGA | TGCCTGTAGTTCCCAAATCA |
| *NDUFA10* | AGGTGGTCGAGGATATTGAG | CTCTGTGAACTCCTGGAAGA |
| *MTCO3* | ATTGGCGGAAGAAGCAGA | GACGGAGTTTACGGCTCAA |
| *ATP5F1A* | CTCTTGAGTCGTGGTGTGCG | CCTGATGTTGGCTGATAACGTG |
| *CYC1* | CCAGGTAGCCAAGGATGTGT | CTTTCGGCTCTTGAGGACTG |
| *DNMT1* | ATTCTCTCCTTCGACACGCC | GCCTTTCAGCTCGCCTTTTC |
| *DNMT3A* | CCTCAGCTCCCCCTACTTATTC | AGCTGTGAGCTTACTCCTGAGC |
| *DNMT3B* | GTTGGTGGCATTGGGATTGT | TTCTCTGGTTGCTTGTTGTTAGG |
| *TET1* | CTTTACTCCAGCCAGCCTCT | GGGTCTGGTAAAGGGGTCTC |
| *TET2* | TCAGTCCAACCCCTCCAATC | ACTGTACCTTGAGACTGCCC |
| *TET3* | AACGGCTTCCACTCCAAGTA | CAAACTCAGCACCACCGTAG |
| *fat-1* | ATTGTCAGGGCGATGTAGGC | CGGCTATCTGGTGTGGAACA |
| *RPLP0* | GGCGACCTGGAAGTCCAACT | GGCGACCTGGAAGTCCAACT |

Supplementary Table 2. The primers of MeDIP-qPCR

| Gene name | Primer sequence | | |
| --- | --- | --- | --- |
|  | Sense (5’to 3’) | Anti-sense (5’to 3’) | |
| *PKM(1)* | TCACGCTCCCGCTCCTAACAC | | AGGGGCTCCGACACAGGAAC |
| *PFKM(2)* | AGACCTGCTCTGGCGGGAAAG | | TCTGGCGGGTGGCCGTTAG |
| *PFKM(3)* | GGCTGCTATGCGAAGGGGTTTC | | CGGTGTTGTGGAGGCGGTTC |
| *NDUFS1(1)* | TTTCTTCGCTGCCTCCCAGTTC | | GTCCGCCAAGTAGTCGTTGAGC |
| *NDUFS1(2)* | GGGCGGAGGGCGTAGCAC | | CTCAACCGACAGGCCACTTCC |
| *ATP5F1C(3)* | AGTGCCGGGACGAGGTAAGC | | ACAGCCCTACTGAGTGGAGTCC |
| *ATP5F1C(4)* | GCTTGCCCTGTTCCGCTTCC | | GCCTTCGGGGTGAGGAAATCTG |
| *IDH2(2)* | TTCCGGGCGCATTAAACCAGTG | | CGCGTAGGAGGATCCGAGAGC |
| *MDH2(4)* | AGCCCGTCTTCTTCCACCTCTC | | AGCCCGTCTTCTTCCACCTCTC |
